# Wide-field imaging in behaving mice as a tool to study cognitive function

**DOI:** 10.1117/1.NPh.11.3.033404

**Published:** 2024-02-19

**Authors:** Ariel Gilad

**Affiliations:** Hebrew University of Jerusalem, Institute for Medical Research Israel-Canada, Department of Medical Neurobiology, Faculty of Medicine, Jerusalem, Israel

**Keywords:** wide-field imaging, behaving mice, cognition, cortical patterns, mesoscale dynamics, working memory

## Abstract

Cognitive functions are mediated through coordinated and dynamic neuronal responses that involve many different areas across the brain. Therefore, it is of high interest to simultaneously record neuronal activity from as many brain areas as possible while the subject performs a cognitive behavioral task. One of the emerging tools to achieve a mesoscopic field of view is wide-field imaging of cortex-wide dynamics in mice. Wide-field imaging is cost-effective, user-friendly, and enables obtaining cortex-wide signals from mice performing complex and demanding cognitive tasks. Importantly, wide-field imaging offers an unbiased cortex-wide observation that sheds light on overlooked cortical regions and highlights parallel processing circuits. Recent wide-field imaging studies have shown that multi-area cortex-wide patterns, rather than just a single area, are involved in encoding cognitive functions. The optical properties of wide-field imaging enable imaging of different brain signals, such as layer-specific, inhibitory subtypes, or neuromodulation signals. Here, I review the main advantages of wide-field imaging in mice, review the recent literature, and discuss future directions of the field. It is expected that wide-field imaging in behaving mice will continue to gain popularity and aid in understanding the mesoscale dynamics underlying cognitive function.

## 1 Introduction

The brain functions as a coordinated organ to mediate cognitive function. To do this, different brain areas constantly communicate with each other, forming a brain-wide network that enables a reliable and dynamic function. Therefore, it is of great interest to simultaneously sample neuronal activity from many brain areas rather than just one area at a time. This brain-wide approach is commonly used in neuroimaging studies (involving humans or animals), where subjects perform complex tasks while their brains are being scanned, e.g., using fMRI.^1^^–^^4^ In short, these studies are mostly limited by both the spatial and temporal resolution and are relatively noisy, making it difficult to track the fast and dynamic networks that may underlie cognitive function. As an alternative, non-human primate studies implemented multi-area electrophysiology^5^^–^^8^ or voltage-sensitive dye imaging^9^^–^^11^ which offered improved spatiotemporal resolution as monkeys performed complex tasks. Nevertheless, the number of simultaneously recorded areas was limited, typically two to six areas (e.g., six visual areas, V4, MT, IT, LIP, PFC, and FEF in Ref. 5).

Another alternative to achieve a mesoscopic field of view is to use the mouse model which offers enormous advantages due to its variety in genetic manipulations. Genetically encoded fluorescent indicators, e.g., calcium indicator, can now be expressed in a specific neuronal type across the whole cortex.^12^ An increasing number of labs have started using wide-field imaging to record population dynamics from the whole dorsal cortex of the mouse, during quiet states,^13^^–^^17^ or during behavior.^14^^,^^18^^–^^22^ Mice are capable of learning difficult tasks, such as memory-related tasks and two-alternative choice tasks, focusing on specific cognitive functions, such as sensory integration, perception, or working memory.^21^^,^^23^^–^^30^ Taken together, wide-field imaging in mice is ideal for studying cortex-wide dynamics underlying cognition. Below, I review the (1) main advantages of wide-field imaging in mice, the (2) biological outcomes from wide-field imaging studies, and (3) discuss future directions of the field. In this review, I will focus mainly on superficial one-photon imaging of the entire dorsal cortex (termed here as wide-field imaging) in mice performing head-fixed cognitive tasks.

## 2 Advantages and Disadvantages of Wide Field Imaging

Wide-field imaging in mice has several advantages making this method suitable to study cognition. Nevertheless, there are also some disadvantages.

### 2.1 Advantages

– Relatively low cost (as low as $1000) and user-friendly;
– Unbiased observation resulting in dynamic cortex-wide patterns rather than single area activity;
– Enables long-term imaging over many days;
– Has a good signal-to-noise ratio enabling single trial analysis;
– Suitable to study the disordered brain;
– Optical-based approach for imaging specific neuronal subtypes or simultaneously imaging different brain signals, or combining optogenetics.

### 2.2 Disadvantages

– Mostly lacking cellular resolution;
– Prone to non-neuronal signals, such as hemodynamics;
– No distinction between different subcellular components, such as axons, dendrites, or cell bodies;
– Does not enable imaging of non-dorsal cortical areas and deep brain structures;
– Head-fixed conditions induce constraints on studying cognitive functions;
– Data analysis can be a bottleneck;
– Produces BIG datasets requiring substantial storage space.

Below I further detail the above points and review the relevant literature.

## 3 Wide-Field Imaging Is Affordable and User-Friendly

Wide-field imaging in mice is a relatively cheap and user-friendly methodology. A typical wide-field setup consists of two major components: an excitation path delivering light to the brain surface and an emission path that projects an image of the light emitted by the fluorescent indicator onto a camera chip. More detailed protocols are described elsewhere.^31^^–^^34^ Importantly, the cost of a wide-field setup is relatively low compared to other imaging setups, ranging from $1000 up to $30,000 depending on the quality of the camera and optics. In addition, a wide-field setup can easily fit within a 70×70  cm bench space, further appealing to small labs.

In terms of mice, a variety of transgenic mice are commercially available and can be used for wide-field imaging.^35^ In many cases, genetically encoded calcium indicators, such as GCaMP6f, can be expressed in either specific neuronal populations (e.g., layer 2/3 excitatory neurons^21^) or more general neuronal populations (e.g., pan-neuronal^36^). Another approach to achieve wide and homogeneous expression is to inject the desired indicator via an injection of AAV vectors into the transverse sinus of neonatal or adult mice.^37^^–^^41^ Today, much progress is being made in developing faster and more sensitive indicators and sensors that will further enhance the spatiotemporal resolution and signal-to-noise ratio of wide-field imaging (Refs. 38, 42, and 43; see Sec. 10).

Several surgical procedures enable full access to the dorsal cortex of the mouse and are described elsewhere.^31^^,^^32^^,^^44^^,^^45^ One appealing and user friendly procedure is the “intact skull” preparation, where the skull is exposed, left intact, and a clear substance is spread on top for protection.^21^^,^^32^ This relatively non-invasive procedure results in a durable and long-lasting preparation, potentially enabling chronic imaging for months. In addition, the surgery itself is rather easy, resulting in a success rate of above 90% even for inexperienced students. The downside is that due to the scattering of light through the skull, the emitted signal lacks cellular resolution. An alternative method that requires more expertise is to replace the skull with a curved cover glass (i.e., crystal skull), gaining cellular resolution even with one photon imaging for several weeks.^44^ In summary, wide-field imaging in behaving mice has become appealing to many labs worldwide, partially due to its low cost and availability of transgenic mouse lines.

## 4 Cortex-Wide Dynamics Underlying Cognitive Function

The main advantage of wide-field imaging is the wide field of view, enabling simultaneous imaging of one or two cortical hemispheres (dorsal part). A mesoscopic view enables access not only to cortical areas that are up an alley of a certain hypothesis but also cortical areas that are initially thought to be unrelated or overlooked.^46^^–^^49^

Many cognitive functions are performed within several seconds, e.g., sensory integration, perception, and working memory. Sensory integration has been studied using wide-field calcium imaging for several different sensory modalities, including whisker tactile,^17^^,^^21^^,^^29^^,^^50^^–^^54^ visual,^22^^,^^55^^–^^64^ auditory,^50^^,^^62^^,^^65^ and olfactory stimulation.^18^ Most studies have found that sensory information is encoded in a widespread manner across the sensory cortex, including lower- and higher-order areas, depending on the sensory modality. In some cases, the sensory information was also found in frontal cortex which is traditionally related to motor function.^57^^,^^61^^,^^64^ In addition, during sensory integration, several areas were found to encode not only information about the stimulus but also higher-order associative information. For example, Gilad et al. imaged the whole dorsal cortex as mice perform a delayed whisker-dependent discrimination task.^21^ They found that whisker-related sensory areas, including barrel cortex (BC), secondary somatosensory cortex (S2), and the rostrolateral association area (RL; part of the PPC), encoded the choice of the mouse independent of texture type, similar to studies using different recording techniques.^66^^–^^69^ Orlandi et al. trained mice on a visual task and found that choice signals were prominent along the ventral visual stream, i.e., lateral association areas, emphasizing the advantage of a mesoscopic field of view.^56^

**Fig. 1:**
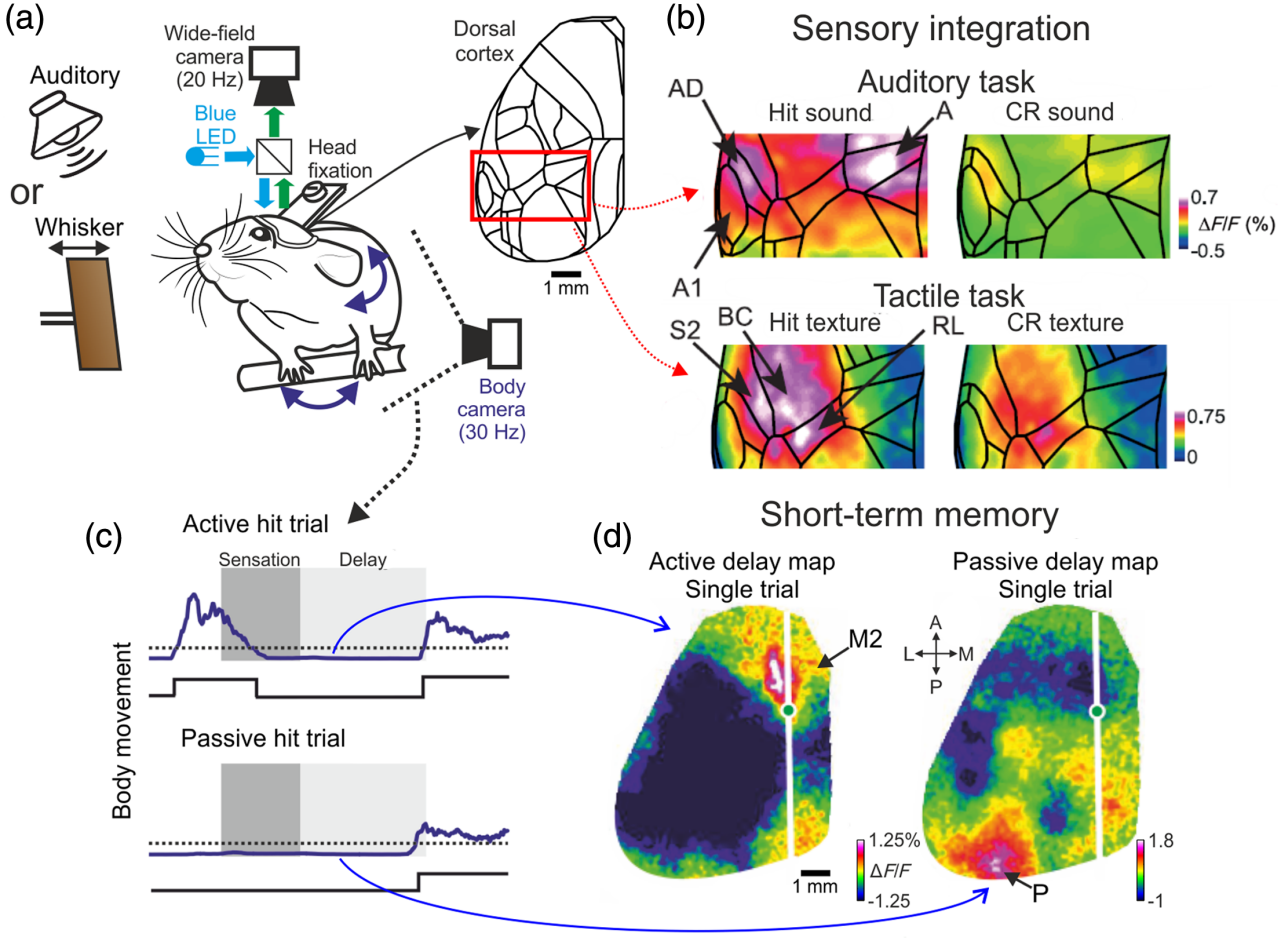
Encoding of cognitive functions across the cortex. (a) Schematic of a wide-field imaging setup for head-fixed mice. Here, the same mouse was trained on a delayed whisker and an auditory task, both with a memory component (i.e., delayed response period). (b) Activity maps averaged during the sensation period for hit and CR trials in whisker or auditory tasks. Only part of the sensory cortex is shown. Auditory and whisker stimulation display distinct activity patches. (c) Body movement was monitored during the task. Example trials of body movements in a whisker task in which the mouse was either active (i.e., moving during the sensation period, top) or passive (i.e., passively waiting for texture approach, bottom). In both cases, the mouse was not moving during the delay period. (d) Cortex-wide activity maps averaged during the delay period during a single active (left) or passive (right) trial. Each map displays distinct activity patterns highlighting M2 for active or P for passive trials. Adapted with permission from Refs. 50 and 21.

One of the advantages of long-term wide-field imaging is emphasized by studies in which mice were trained on several different tasks.^22^^,^^50^^,^^58^ For example, Gallero-Salas et al. trained the same mice on both a whisker-dependent tactile and auditory tasks [Fig. 1(a)] and found that during the sensation period different inter-areal networks encoded each sensory modality [Fig. 1(b); barrel cortex, BC; rostrolateral, RL; secondary somatosensory, S2 for whisker; primary auditory, A1; auditory dorsal, AD; and anterior, A for auditory discrimination; Ref. 50]. This is surprising since association areas, including several PPC areas, are thought to be multisensory, i.e., a single area encodes information from several sensory modalities. Having the ability to concurrently monitor several association areas indicates that in this case, each association area is modality-specific (RL for tactile and A for auditory) rather than multisensory.

Wide-field imaging of the whole dorsal cortex enables large access to both sensory (posterior) and motor (frontal) cortices, making it ideal to study sensorimotor transformation. Several wide-field studies focusing on whisker-dependent tasks were able to highlight several cortical pathways from sensory to motor cortex, e.g., BC to whisker M1, S2 to M2, and RL to M2.^50^^,^^51^^,^^53^^,^^54^^,^^60^ A flow of sensory information from sensory areas to motor cortex was also shown in a visual task.^57^^,^^61^ Once a motor action initiates, it is encoded in a widespread manner that is shown to be dominated by higher-order motor areas, such as M2 and ALM.^19^^,^^57^^,^^60^^,^^61^ To further dissect sensorimotor projection, Matteucci et al. took advantage of optical labeling approaches to image only M2-projecting neurons across the whole dorsal cortex, highlighting an S2 to M2 projection involved in sensorimotor transformation.^53^

Another important cognitive function with relatively fast mesoscale dynamics (several seconds) is short-term memory in which information of a certain stimulus needs to be maintained in memory for several seconds in order to successfully complete a required task. Indeed, in recent years numerous studies used wide-field cortical imaging in mice performing a memory-dependent task.^21^^,^^50^^,^^51^^,^^55^^,^^58^^,^^65^ Wide-field imaging has revealed several cortical areas involved in maintaining short-term memory, including frontal areas, such as M2, and posterior areas, such as areas anterior medial, AM,^58^ and posterior/lateral medial P/LM.^21^^,^^50^ Interestingly, Gilad et al. found that the cortical location of short-term memory varied between activity patches in frontal or posterior cortex depending on the behavioral strategy of each mouse.^21^ If the mouse initiated an active strategy, i.e., moving and whisking toward the incoming texture, short-term memory was located in M2, whereas a passive strategy, i.e., waiting quietly for the incoming texture, elicited activity in area P during short-term memory [Figs. 1(c) and 1(d)]. Importantly, wide-field imaging enabled an unbiased observation of distinct brain-wide patterns during a seemingly identical cognitive behavior. In addition, changing the sensory modality to audition, resulted in similar short-term memory hubs in M2 and P that were dependent on the behavioral strategy.^50^

Esmaeili et al. performed wide-field imaging in mice performing a whisker-dependent task with a delay component.^51^ They found that during the delay period not only did M2 display persistent excitation but also in parallel orofacial sensorimotor cortex displayed transient suppression during the early part of the delay. This study highlights the advantage of having a wide field of view, which enables observation of parallel processes that may have been overlooked in prior studies. Pinto et al. found that during memory-guided navigation, wide-field imaging revealed widespread ramps of activity indicating that neuronal correlates of short-term memory are distributed across several cortical areas.^22^ Widespread cortical recruitment was only present in a memory-guided task and not in a visually guided task that does not carry a memory component. In summary, a majority of studies using wide-field cortical imaging have focused on specific cognitive functions within short time scales. Some have outlined specific areas whereas others have highlighted the spatially distributed encoding of cognitive function. In any case, the outcomes from these types of studies merit further investigation using similar and complementing approaches.

Wide-field imaging is also ideal for processes that prolong for several minutes. For example, different arousal states thought to originate from widespread modulatory effect, have a substantial impact on mesoscale dynamics and the cognitive functions discussed above. Locomotion is another example of a process that is linked to a high arousal state and has been shown to modulate cortex-wide dynamics.^14^^,^^70^^–^^72^ Clancy et al. combined wide-field cortical imaging with single-unit activity in either primary visual (V1) or retrosplenial (RSP) cortices and found that during locomotion, V1 displays higher internal correlations whereas RSP decrease internal correlation and increase inter-areal correlations.^14^ West et al. further found that during steady locomotion, primary motor and somatosensory nodes show decreases in correlations, while retrosplenial and M2 show increased correlations.^72^ Dynamic changes in cortex-wide correlations were also present in the resting state, i.e., sitting quietly, and coincided with changes in arousal state.^70^ To further investigate the relationship between behavioral states and cortex-wide dynamics, Lohani et al. optically separated wide-field signals to simultaneously image both neuronal and acetylcholine (ACh) dynamics across the whole dorsal cortex.^71^ They found that different arousal states correspond to widespread neuronal activity and ACh release in a heterogenic manner. Neuronal activity increased mostly in the posterior cortex during high arousal states associated with locomotion. In contrast, ACh release increased in frontal cortex mainly during a moderate arousal state, associated with high facial movement without locomotion. Jacobs et al. trained mice on either an auditory or a visual task and found slow 3 to 6 Hz fluctuations that were global across the cortex and related more to task engagement (i.e., arousal) rather than accuracy.^62^ Taken together, wide-field imaging can elucidate global and local dynamics that are related to slower fluctuations in behavioral states which may have a strong impact on different cognitive functions.

## 5 Complex Spatiotemporal Dynamics of Wide-Field Imaging

The above studies highlight the power of wide-field imaging in behaving mice. The outcomes of these studies emphasize the distributed nature of cognitive function, present across several different cortical areas, sometimes concurrently. From a mesoscopic point of view, it is of great interest to extract cortex-wide-patterns, rather than single area activation that may underlie different cognitive function. Since wide-field imaging comprises tens of thousands of pixels, the number of possible activity patterns (i.e., an activity snapshot) are infinite, further motivating the field to reduce wide-field signals into lower-dimensional pattern representation. Having said this, cortical activity has its constraints, mainly due to functional and anatomical connectivity and local spread of activity. Several studies have shown cortical motifs that are related to sensory stimulation that also emerge during spontaneous activity.^13^^,^^17^^,^^29^ For example, a whisker stimulation evoked a common cortex-wide activity pattern including whisker-related areas BC, S2, and M1.^13^^,^^17^ These constraints substantially reduce the number of possible observed patterns.

**Fig. 2:**
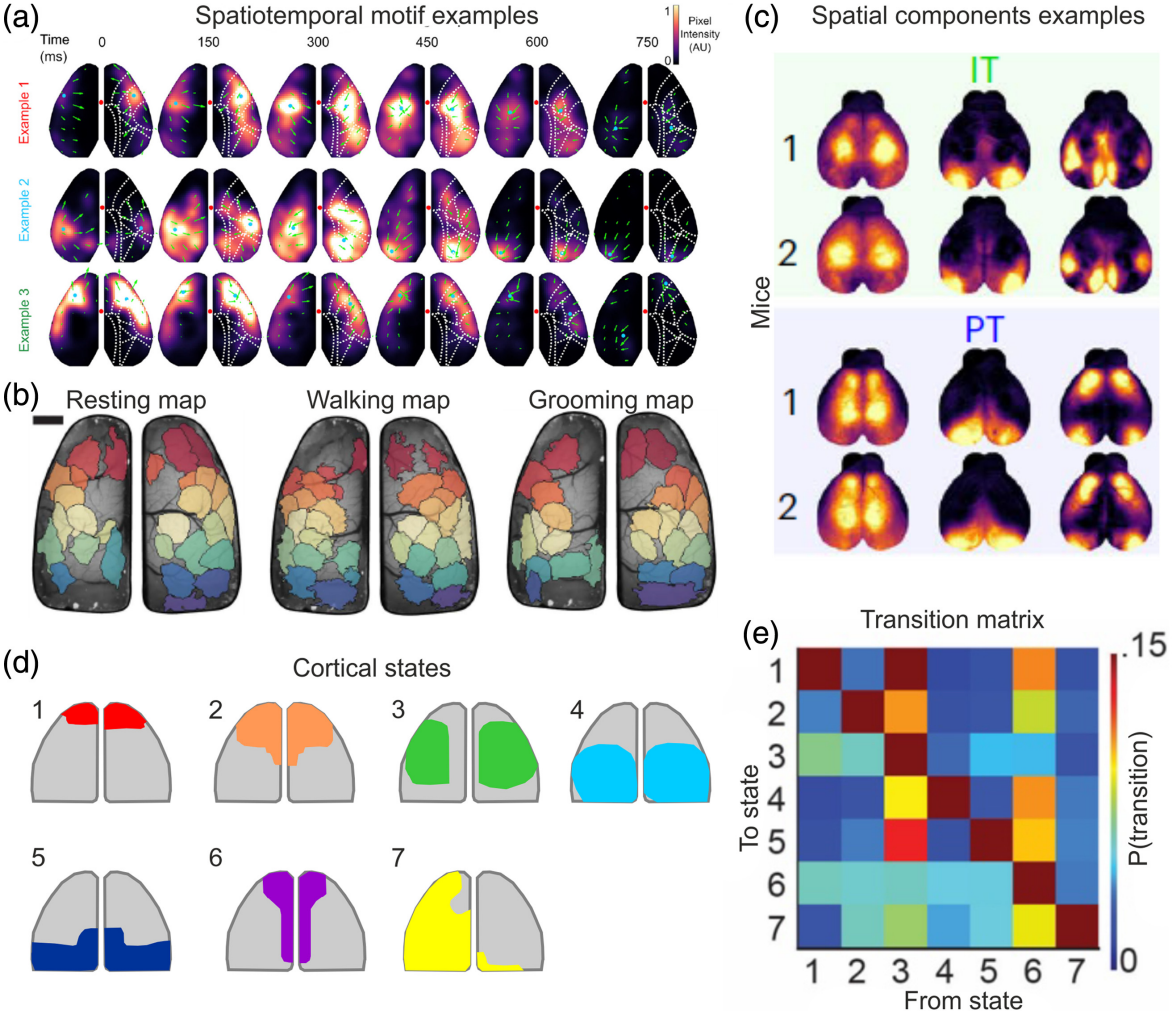
Lower dimensional spatiotemporal patterns from wide-field signals. (a) Three example spatiotemporal cortex-wide motifs during resting state, each highlighting distinct cortical activity patterns. Adapted with permission from Ref. 74. (b) Different spatial ICs (color coded) during resting, walking, or grooming behaviors. There are both common and unique ICs across behaviors. Adapted with permission from Ref. 73. (c) Spatial component examples of different pyramidal neurons (IT or PT, shaded areas) and different mice. Spatial components differ between neuron types but are similar across mice of the same neuron type. Adapted with permission from Ref. 65. (d) Schematic of seven cortical states derived from mice freely navigating a complex maze. (e) The transition matrix between the seven states in d, i.e., the probability from transitioning from one state to another. Adapted with permission from Ref. 76.

There are several data-driven methods that typically produce a limited set of cortex-wide spatiotemporal patterns, e.g., independent component (IC) analysis^19^^,^^72^^,^^73^ or some form of non-negative matrix factorization.^65^^,^^74^^,^^75^ MacDowell et al. found that a set of ∼14 cortex-wide patterns captured most of the wide-field neural dynamics during spontaneous behavior [Fig. 2(a)^74^]. The probability and specificity of these lower-dimensional representations differed across behavioral states, further highlighting their relevance to encoding cognitive functions. Nietz et al. identified a set of spatial ICs that were common across behaviors, but with additional behavior-specific unique ICs [Fig. 2(b)^73^]. Musall et al. imaged cortex-wide dynamics of different types of pyramidal neurons (pyramidal tract, PT, versus intratelencephalic, IT, neurons) as mice perform an auditory task and found that spatial component differs across pyramidal neuron types [Fig. 2(c)^65^]. Furthermore, a simple classifier could discriminate between different types of pyramidal neurons based on these spatial components. Hence, the spatial complexity of wide-field signals can be in some cases reduced to a finite set of spatial patterns.

Although spatial components are an important factor, the temporal dynamics, e.g., the transition between components, may also be critical in understanding cortical function. Surinach et al. performed wide field imaging in freely moving mice navigating a complex maze.^76^ They clustered spatial patterns into 5 to 10 distinct common states during navigation and calculated the transition probability from state to state [Figs. 2(d) and 2(e)]. They found that as mice start navigating toward their goal, a frontal activation pattern emerges and is preceded by a distinct set of spatial patterns that are dependent on the mouse’s search strategy. This study, along with additional upcoming studies, highlights a Markovian nature of wide-field signals. Finally, complex dynamics are not only present in the cortex but also in the complex behavior of the mouse during the task. Using multivariate models, it was shown that movement, arousal, motivational, and different task parameters are highly dynamic within each trial and have a profound effect on cortex-wide dynamics in general and spatial patterns specifically.^21^^,^^53^^,^^55^^,^^60^^,^^77^ These complex interactions between internal (e.g., body movement and arousal) and external (e.g., stimulus type and location) parameters make it challenging to extract cognitive-related signals and require additional caution in the interpretation of wide-field signals. In summary, wide-field signals during behavior are highly complex and dynamic, and in some cases can be represented by lower dimensional patterns.

## 6 Longitudinal Wide-Field Imaging

**Fig. 3:**
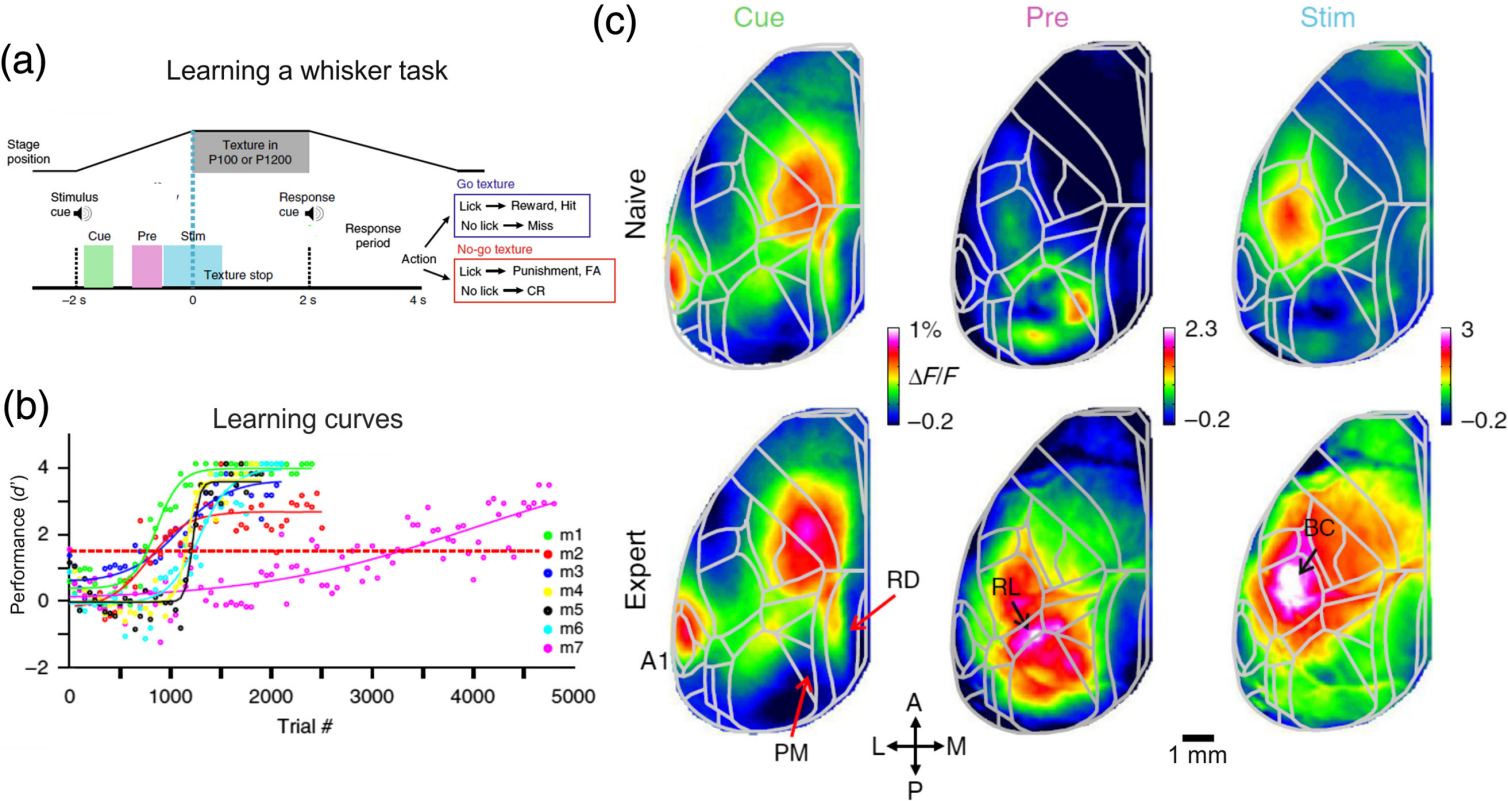
Wide-field imaging during multiple days. (a) Behavioral paradigm for a go/no-go whisker task, highlighting specific temporal periods within the trial (cue, pre and stim periods, different colors). Mice were imaged as they learned the task during 5 to 11 days. (b) Learning curves (i.e., performance as a function of trials) for each mouse separately. Red line indicates learning threshold ($d^{\prime }=1.5$). (c) Cortex-wide activity maps for naïve (top) and expert (bottom) example mouse averaged during the cue, pre, and stim periods. Expert mice display enhanced activity in task-related areas (i.e., A1, RL, and BC) during specific time periods. Adapted with permission from Ref. 29.

Wide-field imaging studies across longer time scales such as hours and days are becoming more and more popular. Day-by-day alignment of wide-field images using blood vessel patterns is easier than tracking identical single cells using, for example, two-photon microscopy. In addition, non-invasive imaging preparations such as the intact skull allow for durable and stable imaging over the course of even several months.^31^^,^^32^ This opens up the opportunity to study longer processes, such as learning. Makino et al. performed longitudinal wide-field imaging during motor learning and found compressed and stable neuronal activity across the cortex, with M2 acquiring a leading role as the mouse gains expertise.^19^ Gilad and Helmchen measured cortex-wide dynamics as mice learn a whisker-dependent discrimination task [Figs. 3(a) and 3(b)^29^]. They find a task-related flow of activity starting from A1 (in response to an auditory cue) to RL (just before texture touch) to BC (during texture touch) which is enhanced during learning [Fig. 3(c)]. Interestingly, other association areas, such as posteromedial (PM) and retrosplenial (RSP) cortices, displayed suppression emerging hundreds of trials before the mouse learned the task. These results indicate that learning induced a widespread refinement of population dynamics in the association cortex. In addition, Marmor et al. found that BC and RL additionally encode trial history, i.e., the choice of the previous trial, only as the mouse learns the task.^78^ History information in RL emerges before texture touch and is thought to transfer past information to BC that integrates past and present information in order to optimize performance.

Ebrahimi et al. used a 5-mm cranial window to simultaneously image 21,000 neurons across eight cortical areas over 5 days in mice performing a visual discrimination task.^59^ They found that encoding of perceptual information remained stable at the population level across the different areas, despite day-to-day variations in the responses of individual cells. In addition, during a short-lived state of ∼300  ms, there was a transient increase in correlated fluctuations among task-related neurons, highlighting the importance of imaging a large-field of view with a good spatiotemporal resolution. The relative stability of wide-field signals further enables to measure cortex-wide signals from the same mouse performing different complex tasks, even several weeks apart.^50^^,^^73^ For example, Gallero-Salas et al. trained the same mice on both a whisker and auditory task requiring several weeks of training for each task.^50^ Sokoletsky et al. trained the same mouse on both a regular and reversed go/no-go whisker to study somatosensory perception. By comparing cortex-wide activity maps from both tasks, they found isolated correlates of perception in medial association cortex.^79^ Nietz et al. found cortex-wide patterns that were stable within seconds to days as mice performed different behaviors, such as resting, walking, and grooming.^73^ On top of common and stable patterns, they also found unique patterns within a short duration that emerged for specific behaviors, highlighting the interplay between stability and flexibility at the mesoscale level. Taken together, these findings bring up an interesting aspect related to long-term computational processing which is representational drift. In short, representational drift describes a phenomenon in which single neurons change their response profile to a certain stimulus (i.e., drift away from their original response) as time goes by.^80^^–^^83^ It is thought that representational drift occurs at the level of single neurons and is averaged out across neurons, resulting in a stable representation at the population level. Whether representational drift is present in wide-field signals is still an open question for future studies. Taken together, wide-field imaging enables longitudinal cortex-wide imaging, giving the opportunity to monitor and compare complex dynamics across many days.

## 7 Wide-Field Imaging Enables Single Trial Analysis

**Fig. 4:**
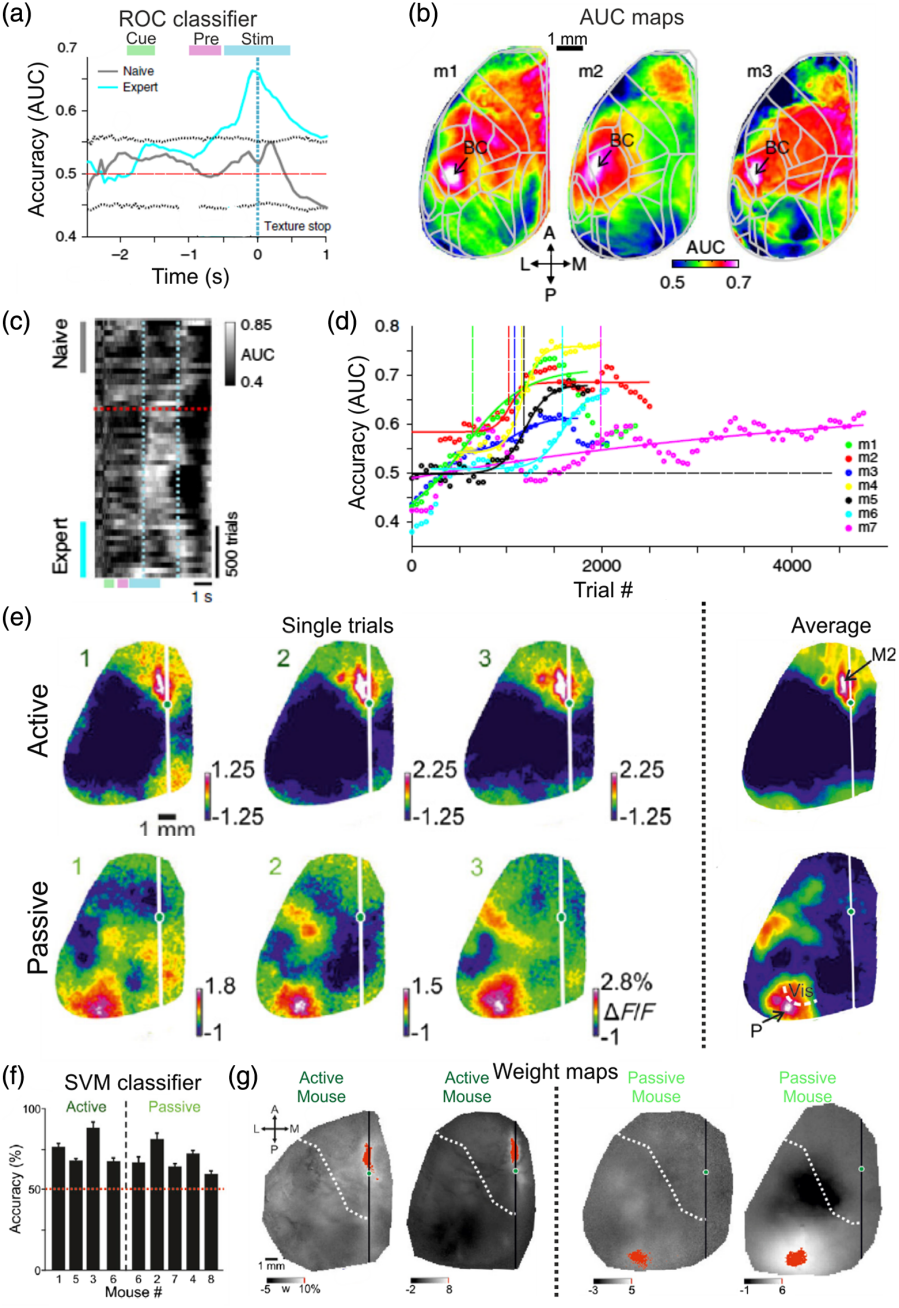
Single trial analysis of wide-field signals. (a) Accuracy of an ROC curve (i.e., area under the curve, AUC) as a function of time between activity in BC during Hit versus CR trials. Expert (cyan) and naïve (gray) cases. Dashed gray lines are mean ± 2 × STD of trial-shuffled data. (b) AUC maps in three example mice averaged during sensation (stim period, blue bar in A). BC displays high accuracy values. (c) AUC values in BC plotted as a function of time (within the trial, x axis) and learning (across days, y axis). BC displays high AUC values mainly during the stim period (blue dashed bars) and after learning (red dashed line). (d) AUC values in BC across the learning profile of different mice displaying similar dynamics as their learning curves presented in Fig. 2(a). (e) Single trial activity maps averaged during the delay period in example active (top) and passive (bottom) trials. On the right is the trial average activity maps. (f) Accuracy of an SVM classifier between hit and CR trials for active or passive mice separately. All mice display significant above chance accuracy. (g) Example weight maps derived by the classifier for active or passive mice. The classifier assigned the highest weights (marked in red) for M2 in active mice and P in passive trials. Adapted with permission from Refs. 29 and 21.

Simply taken, a wide-field signal from a single trial represents the mean neuronal activity of a large number of neurons (considering the possibility of contamination from non-neuronal signal or a mixture of dendritic and axonal signals). This basic spatial averaging enables to observe meaningful activity within a single trial and in some cases single trial activity maps highly resemble the mean activity maps averaged over many trials [Fig. 4(e)]. In contrast, measurements of activity from single neurons are mostly noisy, requiring averaging over many trials in order to get meaningful responses. Reliable signals within single epochs can improve single-trial analyses, e.g., classifying trial types based on cortical dynamics. A rather straightforward single trial classifier is a receiver operating characteristic (ROC) curve, which finds an ideal observer that can classify between two populations. The area under the curve (i.e., AUC) quantifies the accuracy of the classifier, where 1 depicts a perfect classification and 0.5 depicts a random choice. Taking into advantage the reliable wide-field signal, an ROC curve can be implemented both in a pixel-wise manner or for each time frame separately, resulting in a spatiotemporal cortex-wide classification tool.^11^^,^^21^^,^^29^^,^^61^ For example, Gilad and Helmchen imaged the whole dorsal cortex as mice learn a whisker-based go/no-go task.^29^ They calculated the spatiotemporal dynamics of an ROC curve between go and no-go trials and found that discrimination power (i.e., AUC values) increased in BC as the texture approached the whiskers and only after mice became experts [Fig. 4(a)]. Interestingly, classification accuracy across the whole cortex (i.e., AUC maps) emphasizes the high AUC values in BC compared to other cortical areas [Fig. 4(b)]. Furthermore, AUC maps can be calculated for individual mice, highlighting reproducible patterns, but also possible inter-mouse differences that may be of interest [Fig. 4(b)]. In addition, AUC can be calculated for each time frame over the time course of learning, resulting in a two-dimensional temporal profile that indicates the accuracy emergence just after learning [Fig. 4(c)]. Furthermore, AUC values were also calculated as a function of learning, i.e., across days, resulting in an accuracy learning curve for each mouse [Fig. 4(d)]. Taking together, a rather simple classifier can obtain a relatively high accuracy with rich spatiotemporal cortex-wide dynamics.

An ROC analysis is performed separately for each area or pixel, but there could be relevant information that is jointly distributed across the cortex and may be overlooked with a single-pixel classifier. Taking advantage of the simultaneous cortex-wide imaging, there are classifiers that can receive many pixels and find an optimal separation by assigning higher weights to the most informative pixels. In essence, this can result in increased classification accuracy and also highlight spatial patterns that carry relevant information at the single trial level. For example, Gilad et al. used a support vector machine (SVM) to classify hit or correct rejection (CR) trials based on single trial delay maps (i.e., mean activity map during the delay period when the mouse maintained short-term memory).^21^ This was done for an active (i.e., the mouse was moving and whisking during the sensation period) or passive (i.e., the mouse was sitting quietly during the sensation period) strategy separately. Classification accuracy was significant for each mouse and strategy separately [Fig. 4(f)]. Interestingly, when observing the weight maps (i.e., the classifier’s weight for each pixel across the cortex), it is evident that positive weights were assigned to a frontal area (i.e., M2) in the active strategy, whereas positive weights were assigned to a posterior area (i.e., area P) during the passive strategy [Fig. 4(g)]. In another example for single trial analysis, Musall et al. used a linear model to predict single trial dynamics during a visual task and found that activity patterns are mostly dominated by movement parameters of the mouse.^55^ In summary, wide-field cortical imaging enables single-trial analyses that highlight complex processing patterns which are mediated within a single trial.

## 8 Wide-Field Imaging of the Disordered Brain

**Fig. 5:**
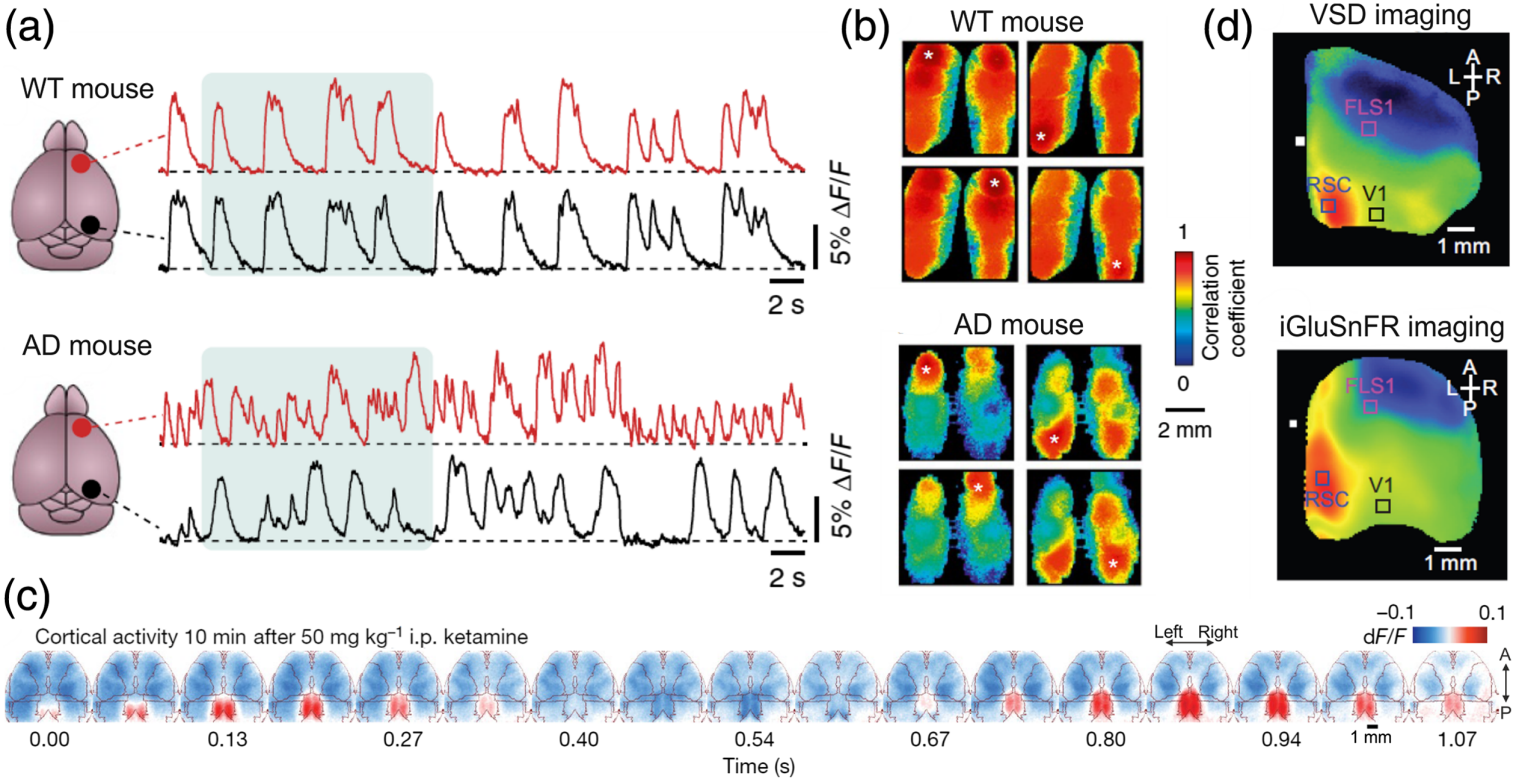
Abnormal cortex-wide patterns. (a) Calcium dynamics from two distant cortical areas from a WT (top) and Alzheimer’s disease (AD, bottom) model mouse. (b) Seed correlation maps (four different seeds marked with a white dot) for WT (top) and AD (bottom) mice. AD mice display asynchronous cortical dynamics. Adapted with permission from Ref. 84. (c) Activity maps as a function of time relative to ketamine injection. RSP displays distinct oscillatory neuronal activity. Adapted with permission from Ref. 85. (d) Average activity map triggered on sharp wave ripples (recorded from the hippocampus) for voltage (top) and glutamate (bottom) during sleep. RSP displays a triggered enhancement. Adapted with permission from Ref. 86.

Wide-field imaging is also optimal for studying plasticity changes that occur over even longer time periods, such as weeks and months. The stability and durability of the methodology, along with available disease-related mouse models, are ideal for studying different neurological disorders and their impact on brain-wide network dynamics. Importantly, one can monitor the progression of network dysfunctions in the same mouse, alongside the development of disease-related symptoms. Although studies using long-term chronic approaches are only starting to take shape, I will describe several studies using wide-field imaging in mouse models for different diseases. Busche et al. used wide-field cortical imaging to study slow wave cortex-wide propagation associated with non-REM sleep in a mouse model for Alzheimer’s disease (AD^84^). They demonstrate that in AD mice, slow-wave activity is severely altered in a cortex-wide manner [Figs. 5(a) and 5(b)]. This mesoscale de-synchronization may cause a breakdown of the characteristic long-range coherence of slow-wave activity which may lead to deficits in memory consolidation. McGirr et al. used voltage-sensitive dye imaging of the whole dorsal cortex to study the effects of stress on spontaneous and sensory-evoked activity.^87^ In a previous study from the lab, they found that during spontaneous activity the cortex transitions between sensory motifs that are similar to sensory-evoked patterns.^13^ Stress (induced using a chronic social defeat protocol) revealed an idiosyncratic increase in spontaneous sensory motifs which is associated with increased variability in sensory-evoked responses. It is implied that changes in sensory processing are linked to unexplained physical complaints in stress-related psychopathology.

Recent studies have used wide-field calcium or voltage imaging to study mesoscale dynamics in mouse models for Huntington disease (HD^88^^,^^89^). Sepers et al. find extensive spread of evoked sensory signals across the cortical surface in HD mice. Alongside the development of high-throughput behavioral tasks for longitudinal monitoring,^89^ it is now possible to monitor behavior and cortical circuit changes in HD for months. Groleau et al. investigated mesoscale cortical dynamics during recovery of optic nerve injury.^90^ Specifically, they were able to track spontaneous and sensory-evoked cortical dynamics before, during, and 31 days after optic nerve crush (ONC) in mice. Cortical responses to visual stimulus decreased in visual areas just after ONC but were partially rescued after 3 to 5 days. In addition, the connectivity between visual and non-visual regions after ONC was decorrelated and recovered only after 31 days. Wide-field imaging could also be used to study epilepsy in an attempt to track local and global spread of epileptic seizures.^91^ In summary, these findings highlight the importance of long-term mesoscale monitoring in studying disease-related abnormalities in the brain.

Another line of studies investigates widespread cortical oscillations during natural sleep and anesthesia, linking mesoscale dynamics to different loss-of-consciousness states.^85^^,^^86^^,^^92^^–^^94^ Vesuna et al. showed that a precisely dosed administration of ketamine or phencyclidine along with wide-field cortical imaging elicited a 1- to 3-Hz rhythm in layer 5 neurons of the retrosplenial cortex [RSP; Fig. 5(c)]. Abadchi et al. measured cortex-wide voltage and glutamate dynamics along with a simultaneous multiunit recording in CA1. They find sharp-wave ripples (SWR) in CA1 are mostly related to neuronal activity in the RSP highlighting a hippocampal-cortical interaction that may underlie memory consolidation during sleep [Fig. 5(d)^86^]. Similar recordings during anesthesia^92^ or awake^95^ reveal distinct and complex mesoscale dynamics related to hippocampal activity, highlighting the advantage of a wide field of view during different consciousness states. Having the ability to measure the dynamics of additional sleep-related neuromodulatory signals in a cortex-wide manner, may further aid in better understanding the brain-wide mechanisms of healthy and disordered sleep.

## 9 Disadvantages of Wide-Field Imaging

There are several disadvantages in wide-field imaging of behaving mice that should be taken into consideration. In most cases, wide-field imaging lacks cellular resolution and measures a bulk signal emitted from a local patch of cortex (volume of several tens on microns). The spatial resolution worsens when imaging through the skull which results in increased scattering of emitted light. To increase spatial resolution, it is possible to implant a full-field cover glass (i.e., crystal skull) over the entire dorsal cortex, or apply substances on top of the skull that make it more transparent.^31^^,^^33^^,^^44^^,^^45^ In addition, the source of the signal is unknown and could originate from different subcellular components (e.g., axons, dendrites, or cell bodies) or also non-neuronal signals, such as hemodynamics.^96^ To overcome this, an additional light (e.g., 405 nm) is interleaved with the regular excitation light (e.g., 473 for GCaMP6) which can control for hemodynamic modulations. Targeting the indicator to only cell bodies can aid in excluding possible signals from axons and dendrites.^97^ In terms of the mice, it has been reported that several GCaMP6 expressing transgenic mouse lines display aberrant cortical activity which can bias the interpretation of the data.^91^ In addition, calcium overexpression can be toxic which can alter neural function.^98^^–^^102^ These issues should be taken under consideration when choosing a specific mouse line.

Wide-field imaging is limited to the dorsal part of the cortex and does not enable imaging of non-dorsal cortical areas that may be of high interest for studying cognition, such as entorhinal, prefrontal, insular, and orbitofrontal cortices. In addition, deeper subcortical areas of interest, such as hippocampus, thalamus, and striatum, are inaccessible when using wide-field imaging (see Sec. 10 for possible solutions). Another disadvantage is that wide-field imaging under head-fixed condition is not ideal to study cognitive functions. In short, head fixation introduces substantial stress and immobility and limits behavioral paradigms to mostly a routine presentation of specific stimuli followed by a stereotypic motor report for a reward. More complex paradigms such as introducing a memory component may expand our ability to study cognition, but require sometimes months of training, making it difficult to maintain stable and reliable imaging. Finally, wide-field imaging produces large datasets that require substantial storage and also complex data analysis. Some of the main challenges in data analysis deal with the high dimensionality of the data, linking signals to complex behavior, and addressing individual differences. Today, there are several open-source data processing tools that aid in coping with the complexities in data analysis, appealing to non-expert users outside the wide-field community.^31^^,^^33^^,^^103^ In summary, wide-field imaging has several disadvantages that can be adequately addressed in some cases.

## 10 Future Directions for Wide-Field Imaging

Wide-field imaging in behaving mice has a bright future. Taking advantage of its optical access along with a variety of transgenic mouse lines,^12^^,^^104^ it is now possible to image cortex-wide dynamics of only a specific neuronal population. For example, layer 2/3 excitatory cells,^21^^,^^29^^,^^50^^,^^53^^,^^58^^,^^59^ layer 5 pyramidal tract neurons,^65^^,^^85^^,^^105^ inhibitory neurons,^18^^,^^106^ and potentially even deeper layer 6 neurons. Wide-field imaging can further measure the cortex-wide dynamics of different neurotransmitters, such as glutamate^86^^,^^95^ and acetylcholine,^71^^,^^94^ opening the field for studying the widespread effects of the diffuse neuromodulatory system. Another promising possibility is to image only projecting specific neurons, e.g., cortex-wide projecting neuron to M2^53^ or widespread axonal terminals in cholinergic neurons.^94^

The development of improved indicators, such as voltage indicators^86^^–^^88^^,^^92^ or faster calcium indicators (e.g., GCaMP8f^42^), will further aid in reliably tracking fast and complex neuronal representations of cognitive functions. A promising direction in the field is to optically separate and simultaneously image different signals (i.e., dual color imaging), e.g., acetylcholine and calcium.^71^ Possible applications include measuring cortex-wide excitation/inhibition balance, neuronal/non-neuronal interactions (e.g., neurons and astrocytes), and multiple neuromodulatory signals (e.g., cholinergic projections from the basal forebrain and noradrenergic projections from the locus coeruleus). These directions may expand our understanding on the mechanistic large-scale dynamics underlying both healthy and disordered cognitive processing.

Cortex is part of a complex and dynamic brain-wide network that mediates cognitive function. Therefore, it is of interest to simultaneously measure from both the whole dorsal cortex and other subcortical areas. Several studies have been able to record electrophysiological signals from subcortical areas, such as the hippocampus^86^^,^^95^^,^^107^ or striatum,^63^ in parallel to wide-field imaging. Multiple tetrodes can be used to target several subcortical areas.^108^ Fiber photometry deeply inserted at an angle from the other hemisphere can also be used to measure population dynamics from subcortical areas while maintaining the advantages of optical signals discussed above.^109^ Future studies can further combine wide-field imaging with multi-fiber photometry^110^ that enables measurement from up to 48 subcortical recording sites. Another imaging method is functional ultrasound, which enables simultaneous measurement of both cortical and subcortical structures in a behaving mouse and is becoming increasingly popular.^111^ An alternative approach is to use other model organisms, such as zebrafish or *Drosophila*, which enable full brain access with cellular resolution, although the ability to study cognition is suboptimal using these models. An interesting future application will be to use patterned optogenetics to manipulate only a subset of subcortical/cortical areas while measuring cortex-wide neuronal dynamics during a cognitive task. Finally, recent advances now enable to mount a wide-field microscope with a cortex-wide field of view on a freely moving mouse^112^ which opens the possibility of tracking cortical dynamics during naturalistic behavior.^76^

In summary, wide-field imaging of behaving mice has become a popular tool that enables cortex-wide measurements of neuronal population dynamics. Wide-field imaging is cost-efficient, user-friendly, and produces reliable and stable signals even within signal trials. Capturing fast mesoscale dynamics will increase our understanding of the brain-wide mechanisms underlying cognition in health and disease.

## Data Availability

Data sharing is not applicable to this article, as no new data were created or analyzed.
